# Utility of Diagnostic Colonoscopy in Pediatric Intestinal Disease

**DOI:** 10.3390/jcm11195747

**Published:** 2022-09-28

**Authors:** Masaru Morita, Hidetoshi Takedatsu, Shinichiro Yoshioka, Keiichi Mitsuyama, Kozo Tsuruta, Kotaro Kuwaki, Ken Kato, Ryosuke Yasuda, Tatsuki Mizuochi, Yushiro Yamashita, Takumi Kawaguchi

**Affiliations:** 1 Division of Gastroenterology, Department of Medicine, Kurume University School of Medicine, 67 Asahi-machi Kurume, Fukuoka 830-0011, Japan; 2 Department of Pediatrics and Child Health, Kurume University School of Medicine, 67 Asahi-machi Kurume, Fukuoka 830-0011, Japan

**Keywords:** pediatric coloscopy, sedation, bowel cleansing preparation, inflammatory bowel disease, complication

## Abstract

Background: The roles and methods of diagnostic colonoscopy in pediatric patients were previously demonstrated. With advances in medical equipment and the increasing need for pediatric endoscopic diagnosis, we compared recent results with those previously reported. Methods: A retrospective analysis was conducted on pediatric patients aged ≤15 years, comparing those who underwent their first diagnostic colonoscopy between 1 January 2007 and 28 February 2015 with those who did so between 1 March 2015 and 28 February 2022 at Kurume University Hospital. Results: A total of 274 patients were included, including 110 in the previous study and 164 in the present study. The main indications were hematochezia in the previous study (63/110, 57.3%) and abdominal pain in the present study (64/164, 39.0%). Ulcerative colitis (74/274, 27.0%) was the most common diagnosis in both studies. The major difference from the previous study was an increase in the number of Crohn’s disease and eosinophilic gastrointestinal disorder cases. Bowel preparation with magnesium citrate was significantly increased across all ages in the present study (142/164, 86.6%). Midazolam + pentazocine was used for sedation in most cases (137/164, 83.5%). An ultrathin upper endoscope was mainly used in patients aged ≤6 years, while ultrathin colonoscopes were applied in patients aged 7–12 years. Conclusion: In the present study, appropriate changes were found in the roles and methods of diagnostic colonoscopy in pediatric patients compared to the previous study. The increasing trend of patients presenting with inflammatory bowel disease and eosinophilic gastrointestinal disorder worldwide indicates the importance of colonoscopy in infants and children.

## 1. Introduction

With the development of endoscopes with improved size and flexibility, colonoscopy has become possible, which has led to an increased number of colonoscopies as a diagnostic and therapeutic tool for pediatric patients [1]. However, pediatric colonoscopy has a greater risk of serious complications due to technical difficulties, low compliance with bowel cleansing, and uncooperativeness during the procedure. The largest safety report was a 5-year retrospective database study of 7792 procedures with an overall complication rate of 1.1%, of which approximately 50% were gastrointestinal-related, most commonly bleeding, while 30% were cardiopulmonary complications [2]. Therefore, suitable bowel-cleansing preparation, appropriate sedation for painlessness and safety, and the choice of an appropriate endoscope are very important for performing a reliable pediatric colonoscopy.

According to the increased need for endoscopy in infants and children, the guidelines for colonoscopy in pediatric patients were published by the American Society for Gastrointestinal Endoscopy (ASGE) and the North American Society for Pediatric Gastroenterology, Hepatology, and Nutrition (NASPGHAN), highlighting the indications in children [3,4]. Furthermore, the European Society for Pediatric Gastroenterology, Hepatology, and Nutrition and the European Society of Gastrointestinal Endoscopy published a comprehensive review of the clinical indications and timing of diagnostic and therapeutic endoscopy in pediatric patients in 2017 [5]. In children, suspected inflammatory bowel disease (IBD); per rectal bleeding; unexplained anemia; and genetic polyposis syndromes, including familial adenomatous polyposis, are primarily indicated for pediatric colonoscopy examination [6,7,8]. Despite the importance of diagnostic colonoscopy in infants and children, few colonoscopies that were performed in detail have been reported.

In 2017, we reported the following: (1) patient clinical characteristics, (2) bowel-cleansing preparation, (3) sedation, (4) the choice of endoscope, and (5) the diagnostic utility of colonoscopy in pediatric patients from 2007 to 2015 [9]. In this study, with advances in medical equipment and the increasing need for pediatric endoscopic diagnosis, including IBD, eosinophilic gastrointestinal disorder, and polyposis in recent years, the results of pediatric coloscopies performed at our facility from 2015 to 2022 were compared with those previously reported to investigate the optimization of colonoscopy procedures in infants and children.

## 2. Materials and Methods

### 2.1. Study Protocol and Data Collection

A retrospective analysis was conducted on pediatric patients aged ≤15 years, comparing those who underwent their first diagnostic colonoscopy, except for repeated colonoscopies, between 1 January 2007 and 28 February 2015 with those who did so between 1 March 2015 and 28 February 2022, at Kurume University Hospital. Colonoscopies were performed in children after clinical evaluation by pediatric gastroenterologists. Endoscopic procedures were performed by four advanced experienced endoscopists. The present study protocol was approved by the Human Ethics Committee of Kurume University School of Medicine (approval No. 22076).

### 2.2. Bowel-Cleansing Preparation

Bowel-cleansing preparations for colonoscopy were selected on the basis of age, body weight, and clinical state by pediatric gastroenterologists. Magnesium citrate (Magcorol), polyethylene glycol (PEG) 4000, and glycerin enema (GE), which are licensed for bowel-cleansing preparation in Japan, were used.

### 2.3. Sedation

Sedation methods were selected at the discretion of pediatric gastroenterologists. Thiamylal, the combination of thiamylal and pentazocine, midazolam, and the combination of midazolam and pentazocine were used for sedation in pediatric patients. These sedatives were administered on a weight-based dose, and additional sedatives were added as needed for efficacy. Continuous pulse oximetry and heart rate monitoring were performed throughout sedation to monitor the patient’s vital signs.

### 2.4. Endoscopes

Endoscopes were selected on the basis of patient age and body size by endoscopists. Carbon dioxide was used during the colonoscopy. Mucosal biopsies were performed according to the presence of macroscopic abnormalities and endoscopist experience.

### 2.5. Statistical Analyses

All data were summarized and presented as the mean  ±  standard deviation for continuous variables. We considered *p*-values < 0.05 as statistically significant. Statistical analyses were performed using JMP^®^ Pro 16.0 (SAS Institute; Cary, NC, USA) software. Categorical variables were compared using a chi-squared test.

## 3. Results

### 3.1. Characteristics of Pediatric Patients Who Underwent Colonoscopy

Children aged ≤15 years who were referred for gastrointestinal symptoms with an indication for diagnostic colonoscopy were recruited between 1 January 2007 and 28 February 2015 (previous study), and between 1 March 2015 and 28 February 2022 (present study) (Table 1). A total of 274 patients, including 110 patients in the previous study and 164 patients in the present study, were prepared for their first diagnostic colonoscopy by pediatric gastroenterologists. There were six patients aged <1 year, 28 patients aged 1–3 years, 31 patients aged 4–6 years, 35 patients aged 7–9 years, 77 patients aged 10–12 years, and 97 patients aged 13–15 years. The number of colonoscopies per year increased approximately twofold compared to the previous study. A total of 105 out of 110 patients (95.2%) in the previous study and 161 out of 164 patients (98.2%) in the present study achieved terminal ileum incubation. Hematochezia was the most common symptom in the previous study (63/110, 57.3%), while abdominal pain was the most common indication for colonoscopy in the present study (64/164, 39.0%), with an increased number of cases. A definitive diagnosis was obtained through colonoscopy in 70.9% (78/110) of patients in the previous study and in 64.0% (105/164) of patients in the present study. IBD, comprising ulcerative colitis (UC) (37/164, 20.7%) and Crohn’s disease (CD) (28/164, 17.1%), was the most common diagnosis in both studies. The major difference from the previous study was the increased number of CD, inflammatory bowel disease unclassified (IBDU), and eosinophilic gastrointestinal disorder (EGID) cases in the present study.

### 3.2. Bowel-Cleansing Preparation and Sedation

Table 2 shows the preparation methods used for bowel cleansing. Bowel-cleansing preparation protocols for pediatric colonoscopy were selected by pediatric gastroenterologists. In the previous study, 74 of 110 (67.3%) patients, especially the majority of patients aged ≤12 years (63/79, 79.7%), used Magcorol. Patients aged 13–15 years were treated with PEG-4000. However, Magcorol preparation was significantly increased in patients aged 13–15 years and overall in the present study (142/164, 86.6%).

Sedation was used in 85 patients (77.3%) in the previous study, whereas almost all patients in the present study used sedation (163/164, 99.4%) (Table 3). In the previous study, thiamylal + pentazocine was more frequently used in patients aged ≤6 years (12/28, 42.9%); however, midazolam + pentazocine was used in most cases except for infants in the present study. Despite the change in medication for sedation, no complications, such as hypoxia or allergy, occurred during the colonoscopy procedure.

### 3.3. Colonoscopes Used in Pediatric Patients

Three types of endoscopes, namely, 1030 mm, 1100 mm, and 1330 mm, are used in our facility (Table 4). Upper endoscopy was previously used (8/28, 28.6%); however, ultrathin upper endoscopes were used more often in patients aged ≤6 years in the present study (25/37, 73.0%). Colonoscopes with a length of 1330 mm were used in almost all patients aged ≥7 years in both previous and present studies. Furthermore, endoscopes with eight different shaft diameters were used (Table 5). In the present study, an ultrathin endoscope with a diameter of 5.4 mm was mainly used in patients aged ≤6 years (25/37, 67.6%). Additionally, the PCF-PQ (Olympus, Tokyo, Japan) endoscope with a diameter of 9.2 mm, which is ultrathin and designed for colonoscopy, was used in patients aged 7–9 and 10–12 years (16/16, 100% and 25/45, 55.6%). No serious complications, such as bleeding or perforation, occurred during the colonoscopy. Therefore, endoscopes were appropriately selected by endoscopists according to the patient’s body size and age.

### 3.4. Association between Symptoms and Final Diagnoses

Hematochezia was a symptom with a high positive diagnosis rate (100/117, 85.5%), including UC (63/117, 53.8%) and juvenile polyp (JP) (16/117, 13.7%). Diagnoses of UC were frequently observed in both previous (34/63, 54.0%) and present studies (29/54, 53.7%). Abdominal pain was the most common reason for colonoscopy in the present study, in contrast to hematochezia in previous studies (Table 1 and Table 6). The positivity rate of diagnosis was less than half (30/64, 46.9%), although many pediatric patients underwent diagnostic colonoscopy due to abdominal pain. However, CD (12/64, 18.8%), IBDU (3/64, 4.7%), and EGID (6/64, 9.4%) were included among the cases with abdominal pain. Compared to the previous study, significant increases in CD, IBDU, and EGID were observed in the present study (*p* = 0.028).

## 4. Discussion

Colorectal cancer rarely occurs in children, and colonoscopy is considered an invasive procedure for pediatricians; thus, the number of pediatric colonoscopies at the population level is lower than that of adults. Therefore, colonoscopy is not performed at the appropriate time, resulting in delayed diagnosis and disease progression. With the increasing number of patients with IBD, achieving 100% of terminal incubations is considered important [10]. The registry of newly diagnosed IBD cases in children in 44 centers in 18 countries reported successful ileum intubation in 75% of 1995 colonoscopies [11]. Nambu et al. showed that the terminal ileum was reached in 62% of the diagnostic colonoscopies because of severe colitis, poor bowel preparation, and technical failure [12]. In our facility, a high intubation rate into the terminal ileum, 95.5% in the previous study and 98.2% in the present study, was achieved. This is considered important to the established cooperative system in which pediatricians determine the indications for colonoscopy, bowel preparation, and management of sedation, while gastroenterologists, who specialize in endoscopy, perform colonoscopies.

Bowel preparation regimens for children have not been standardized and vary among medical centers. The majority of prospective and comparative studies of bowel preparation for pediatric colonoscopy have been performed at single centers [13,14,15]. A wide variety of laxatives (including oral and suppositories) are used for bowel preparation; however, evidence to determine the superiority of each drug remains insufficient. PEG is the most commonly used bowel-cleansing agent for bowel preparation regimens in children. PEG-4000 and Magcorol are routinely used for children in most medical centers in Japan. However, the use of Magcorol for bowel preparation has increased with respect to PEG, even in patients aged ≥13 years. In our facility, many pediatric patients could not accept PEG because of the taste and smell; thus, bowel preparation was mainly performed with Magcorol.

Studies for sedation in pediatric gastrointestinal endoscopy have reported several drug combinations, including midazolam- and propofol-containing regimens. Regimens containing propofol are more effective, which provide adequate sedation in 99.9% of cases. Van Beek et al. demonstrated in a systematic review that the propofol-containing regimen is superior to midazolam and opioid analgesics in terms of sedation efficacy [16]. Unfortunately, propofol is rarely used in Japan because pediatric colonoscopy is performed without the presence of an anesthesiologist. Meanwhile, the combination of midazolam with meperidine [17], fentanyl [18], and ketamine [19] is also associated with high completion rates of 91.6–100% without serious side effects. In our facility, midazolam and thiamylal were administered alone or in combination with pentazocine. In a previous study, thiamylal was used in more than half of the pediatric patients aged ≤6 years (15/28, 53.6%). However, increasing the evidence on the efficacy and safety of midazolam, midazolam-based sedation was used in most pediatric patients aged ≤6 years in the present study (32/37, 86.5%). The use of adequate midazolam-based sedation in pediatric patients is necessary to safely perform diagnostic colonoscopy.

Major differences between pediatric and adult colons are their length and diameter. Adult colonoscopes (11.7–13 mm diameter) are acceptable in teenage patients approaching adult size [20]. Smaller, more flexible colonoscopes (11.7 mm diameter) are suitable for preschool- and elementary school-age children [21]. Standard adult upper endoscopes are used for colonoscopy in infants and toddlers [22]. The guidelines of colonoscopy for pediatric patients provided by ASGE and NASPGHAN recommend the use of endoscopes that are smaller than 6 mm in diameter in infants and children weighing <10 kg [4]. In the present study, ultrathin upper endoscopes were used in patients aged ≤6 years (25/37, 73.0%), and ultrathin colonoscopes were used for patients aged 7–9 and 10–12 years (16/16, 100% and 25/45, 55.6%, respectively). According to the improvement of endoscopic image quality, our study showed that ultrathin endoscopes provided appropriate observation and diagnosis.

Common inductions for pediatric colonoscopy include chronic diarrhea, hematochezia, unexplained anemia, polyposis syndrome, and failure to thrive/weight loss [4]. Lei et al. reported that hematochezia, abdominal pain, and diarrhea were the most common reasons for pediatric colonoscopy, as also indicated in our study [23]. Compared with adults, pediatric patients are reported to have a higher frequency of positive findings resulting from colonoscopy [7,23,24]. Among the patients, 65.5% (173/264) had a positive diagnosis, including IBD, EGID, and JP. In pediatric patients, several studies have reported an increased incidence of pediatric IBD and EGID [6,8,12]. In the present study, UC and JP were presented with hematochezia, while CD and EGID were presented with abdominal pain. More than half of the patients presenting with abdominal pain had negative findings, but this is useful in diagnosing important diseases, such as CD and EGID, which have been increasing in recent years [25]. The small intestine is also involved in these diseases; thus, observing the terminal ileum is necessary. Our present study results indicate that colonoscopy can be utilized as a diagnostic tool for pediatric patients presenting with hematochezia, abdominal pain, or diarrhea.

This study had certain limitations. Firstly, this was a single-center and retrospective observational study. Secondly, we did not collect several data, such as cleansing scores in bowel preparation; the degree of sedation and pain during the procedure of colonoscopy; and height, body weight, and BMI for selection of the colonoscope. Future studies will be conducted to include these data in a multicenter and prospective study.

## 5. Conclusions

The present study investigated the role and methods of diagnostic colonoscopy in infants and children. The most common diseases diagnosed by colonoscopy in pediatric patients were IBD, EGID, and JP. Compared to the previous study, an increased number of CD and EGID cases were found. The increasing trend of patients presenting with IBD and EGID worldwide indicates the importance of the role of colonoscopy in infants and children in the future.

## Figures and Tables

**Table 1:** Characteristics of Pediatric Patients.

		Previous Study	Present Study	Total
Total number of patients		110	164	274
Number of patients/year		13.5	23.4	18.1
Terminal incubation (%)		105 (95.5%)	161 (98.2%)	268 (97.8%)
Age (years)	0 1–3 4–6 7–9 10–12 13–15	3 14 11 19 32 31	3 14 20 16 45 66	6 (2.2%) 28 (10.2%) 31 (11.3%) 35 (12.8%) 77 (28.1%) 97 (35.4%)
Gender	Male Female	61 49	89 75	150 (54.7%) 124 (45.3%)
Reason for coloscopy	Hematochezia Abdominal pain Diarrhea Constipation Anemia Anal fistula Genital ulcer Other	63 20 19 0 2 2 2 2	54 64 23 3 1 6 0 13	117 (42.7%) 84 (30.7%) 42 (15.3%) 3 (1.1%) 3 (1.1%) 8 (2.9%) 2 (0.7%) 15 (5.5%)
Diagnosis	Ulcerative colitis Crohn’s disease IBD unclassified EGID No specific colitis Juvenile polyp Normal Other	37 12 0 0 13 7 32 9	37 28 5 6 10 9 59 10	74 (27.0%) 40 (14.6%) 5 (1.8%) 6 (2.2%) 23 (8.4%) 16 (5.9%) 91 (33.2%) 19 (6.9%)

IBD unclassified: inflammatory bowel disease unclassified; EGID: eosinophilic gastrointestinal disorder.

**Table 2:** Bowel-Cleansing Preparation.

Age	*n*	Previous Study (*n* = 110)	Present Study (*n* = 164)	*p*
		Magcorol, PEG, GE, Unknown		
0	6	2, 0, 1, 0	0, 0, 3, 0	0.392
1–3	28	10, 1, 1, 2	13, 0, 1, 0	0.335
4–6	31	9, 0, 1, 1	19, 0, 1, 0	0.544
7–9	35	15, 2, 1, 1	15, 0, 1, 0	0.429
10–12	77	27, 4, 1, 0	43, 2, 0, 0	0.375
13–15	97	11, 20, 0, 0	52, 13, 1, 0	<0.001
Total	274	74, 27, 5, 4	142, 15, 7, 0	<0.001

Magcorol: magnesium citrate; PEG: polyethylene glycol; GE: glycerin enema.

**Table 3:** Sedation.

Age	*n*	Previous Study (*n* = 110)	Present Study (*n* = 164)	*p*
		Thi, Thi + Pen, Mid, Mid + Pen, All, No, Unknown		
0	6	0, 1, 0, 2, 0, 0, 0	1, 2, 0, 0, 0, 0, 0	0.766
1–3	28	1, 7, 1, 3, 0, 0, 2	0, 2, 0, 6, 6, 0, 0	0.032
4–6	31	1, 4, 0, 4, 1, 0, 1	0, 0, 0, 17, 3, 0, 0	0.035
7–9	35	0, 2, 0, 15, 0, 0, 2	0, 1, 0, 15, 0, 0, 0	0.911
10–12	77	1, 4, 2, 21, 0, 4, 0	0, 0, 0, 41, 4, 0, 0	<0.005
13–15	97	0, 1, 1, 13, 0, 16, 0	0, 2, 2, 58, 3, 1, 0	<0.001

Thi: thiamylal; Pen: pentazocine; Mid: midazolam; All: thiamylal + midazolam + pentazocine; No: none.

**Table 4:** Length of endoscopes.

Age	*n*	Previous Study (*n* = 110)	Present Study (*n* = 164)	*p*
		1030, 1100, 1330 mm		
0	6	0, 3, 0	0, 3, 0	1.0
1–3	28	6, 5, 3	0, 12, 2	0.011
4–6	31	2, 0, 9	0, 10, 10	0.058
7–9	35	0, 0, 19	0, 0, 16	1.0
10–12	77	0, 0, 32	0, 1, 44	0.698
13–15	97	0, 0, 31	0, 0, 66	1.0
Total	274	8, 8, 94	0, 26, 138	<0.001

**Table 5:** Shaft diameter of endoscopes.

Age	*n*	Previous Study (*n* = 110)	Present Study (*n* = 164)	*p*
		5.4, 9.2, 9.8, 10.5, 11.3, 11.7, 13.2, 14.8 mm		
0	6	3, 0, 0, 0, 0, 0, 0, 0	3, 0, 0, 0, 0, 0, 0, 0	1.0
1–3	28	5, 5, 2, 1, 1, 0, 0, 0	12, 2, 0, 0, 0, 0, 0, 0	0.444
4–6	31	0, 3, 1, 1, 5, 1, 0, 0	10, 10, 0, 0, 0, 0, 0, 0	<0.005
7–9	35	0, 1, 0, 1, 14, 3, 0, 0	0, 16, 0, 0, 0, 0, 0, 0	<0.001
10–12	77	0, 0, 0, 2, 21, 9, 0, 0	1, 25, 0, 0, 0, 19, 0, 0	<0.001
13–15	97	0, 1, 0, 0, 17, 9, 1, 3	0, 10, 0, 0, 0, 56, 0, 0	<0.001
Total	274	8, 10, 3, 5, 58, 22, 1, 3	26, 63, 0, 0, 0, 75, 0, 0	<0.001

**Table 6:** Symptoms and Final Diagnosis.

Symptoms	*n*	Previous Study (*n* = 110)	Present Study (*n* = 164)	*p*
		UC, CD, IBDU, EGID, NSC, JP, N, Other		
Hematochezia	117	34, 1, 0, 0, 7, 7, 9, 5	29, 0, 1, 0, 5, 8, 8, 3	0.913
Abdominal pain	84	1, 6, 0, 0, 2, 0, 10, 1	1, 12, 3, 6, 4, 0, 34, 4	0.679
Diarrhea	42	1, 4, 0, 0, 3, 0, 10, 1	6, 10, 1, 0, 0, 0, 6, 0	0.105
Constipation	3	0, 0, 0, 0, 0, 0, 0, 0	0, 0, 0, 0, 0, 0, 2, 1	1.0
Anemia	3	1, 0, 0, 0, 0, 0, 1, 0	0, 0, 0, 0, 0, 1, 0, 0	0.885
Anal fistula	8	0, 1, 0, 0, 1, 0, 0, 0	0, 4, 0, 0, 1, 0, 1, 0	0.998
Genital ulcer	2	0, 0, 0, 0, 0, 0, 0, 2	0, 0, 0, 0, 0, 0, 0, 0	1.0
Other	15	0, 0, 0, 0, 0, 0, 2, 0	1, 1, 0, 0, 1, 0, 9, 1	0.997
Total	274	37, 12, 0, 0, 13, 7, 32, 9	37, 27, 5, 6, 11, 9, 60, 9	0.028

UC: ulcerative colitis; CD: Crohn’s disease; IBDU: inflammatory bowel disease unclassified; EGID: eosinophilic gastrointestinal disorder; NSC: nonspecific colitis; JP: juvenile polyp; N: normal.

## Data Availability

Not applicable.
